# Prevalence and Associated Factors of Diabetes Distress, Depression and Anxiety Among Primary Care Patients With Type 2 Diabetes During the COVID-19 Pandemic in Egypt: A Cross-Sectional Study

**DOI:** 10.3389/fpsyt.2022.937973

**Published:** 2022-06-03

**Authors:** Hazem A. Sayed Ahmed, Ahmed Mahmoud Fouad, Sally Fawzy Elotla, Anwar I. Joudeh, Mona Mostafa, Asghar Shah, Jaffer Shah, Samar F. Mohamed

**Affiliations:** ^1^Department of Family Medicine, Faculty of Medicine, Suez Canal University, Ismailia, Egypt; ^2^Department of Public Health, Occupational and Environmental Medicine, Faculty of Medicine, Suez Canal University, Ismailia, Egypt; ^3^Department of Internal Medicine, Hamad Medical Corporation, Doha, Qatar; ^4^Department of Internal Medicine, Faculty of Medicine, University of Jordan, Amman, Jordan; ^5^Department of Internal Medicine, Faculty of Medicine, Suez Canal University, Ismailia, Egypt; ^6^Division of Biology and Medicine, Brown University, Providence, RI, United States; ^7^Medical Research Center, Kateb University, Kabul, Afghanistan

**Keywords:** anxiety, COVID-19, depression, diabetes distress, primary healthcare, type 2 diabetes

## Abstract

The prevalence of type 2 diabetes mellitus (T2DM) is growing worldwide. T2DM is often complicated by a range of psychological disorders that interfere with glycemic control and self-care. Previous studies have reported diabetes distress, depression, and anxiety among patients with T2DM; however; little is known about the burden of these comorbid mental disorders in primary care patients with T2DM treated in Egypt during the COVID-19 era. Participants were selected by convenient sampling from eight rural primary healthcare facilities from Ismailia in Egypt. Symptoms of diabetes distress, depression and anxiety were assessed by using the Arabic version of the 20-item Problem Areas in Diabetes (PAID), Patient Health Questionnaire 9, and Generalized Anxiety Disorder Scales, respectively. Multiple hierarchical logistic regression models were used to estimate the significant factors associated with diabetes distress, depression, and anxiety. A total of 403 individuals with T2DM were interviewed. The prevalence of severe diabetes distress was 13.4% (95% CI: 10.1–16.7), while prevalence of depressive and anxiety symptoms was 9.2% (95% CI: 6.4–12.0%), and 4.0% (95% CI: 2.1–5.9), respectively. In a series of hierarchical logistic regression models, significant predictors for diabetes distress were being married, illiterate, not-working, living with insufficient income, and having multi-comorbidities. Likewise, the significant predictors for depression and anxiety were elevated glycated hemoglobin level and the higher PAID total score, while having multi-comorbidities was a significant predictor for anxiety only. Diabetes distress was more prevalent than depressive and anxiety symptoms in this study population. Several sociodemographic and clinical characteristics were identified to be related with psychological problems among patients with T2DM, which necessitate a multidisciplinary team-based approach for optimal screening and management.

## Highlights

- Diabetes distress was more prevalent than depression and anxiety symptoms among patients with type 2 diabetes at rural primary healthcare facilities.
- The significant predictors for diabetes distress were being married, illiterate, not working, living with insufficient income, and having multi-comorbidities.
- The significant predictors for depression and anxiety symptoms were elevated HbA1c level and the higher PAID score, while having multi-comorbidities was a significant predictor for anxiety symptoms only.

## Introduction

Diabetes mellitus (DM) is a widespread global health problem. Egypt has the 10th highest age-adjusted diabetes prevalence globally, with a comparative diabetes prevalence of 20.9% in people aged 20–79 years. T2DM is the most common form of DM, accounting for 90% of all cases of DM worldwide. T2DM can lead to premature death, a wide range of psychological disorders and decreased quality of life. Additionally, T2DM poses an economic burden on patients, families, and countries (1). DM increases the risk of contracting COVID-19 infection, the risk for hospitalization or intensive care unit admission, and the risk for death (2–4).

The management of patients with T2DM is often complicated by a breadth of psychological disorders including diabetes distress (DD), depression, and anxiety which might negatively impact diabetic patients' quality of life and coping mechanisms with their disease (5, 6). The era of COVID-19 represents a special situation where external stressors, economic burden, risk of acquiring the infection or limited access to healthcare could endanger the mental health of patients with chronic illnesses including those with diabetes (7). A cross-sectional study on 120 patients with type 1 and type 2 diabetes mellitus in Brazil during the COVID-19 pandemic found that more than 90% of the participants had features of ongoing mental suffering and around 40% had significant psychological distress with a higher tendency in patients with T2DM (8).

DD is the emotional burden associated with DM and its management over time (9, 10). A previous meta-analysis demonstrated that the overall prevalence of DD globally was 36% (11). The prevalence of DD in primary healthcare (PHC) patients with T2DM has been reported less than the prevalence among those treated in secondary care (12). Its prevalence among PHC patients with T2DM at primary care level varied across countries; it was 1.2% in Germany, 4% in the Netherlands, 8.9% in Thailand, 9.3–21% in the United States, 22.3% in Saudi, and 24.4% in Greece (12–18). Higher levels of DD are linked with lower quality of life, elevated glycated hemoglobin level (HbA1c), and decreased glycemic control among T2DM patients (19–21).

Depressive symptoms are important indicators in individuals with T2DM. DD and depression are correlated and partly overlapping constructs but are not interchangeable (22). One meta-analysis demonstrated that the prevalence estimates of depression among individuals with T2DM in low and middle-income countries ranged from 25 to 45% with an average of 35.7%. These estimates were significantly higher than estimates in high-income countries, which had a 25% prevalence of co-morbid depression (23). The prevalence rates of depression among individuals with T2DM managing in PHC settings were 11.5 to 26.6% in Malaysia (24, 25), 17% in the United Arab Emirates (26), 20 to 37.9% in Saudi Arabia (17, 27–29), 40.2% in Palestine (30), 20.03 to 29.2% in Spain (31, 32), 30.3% in Germany (33), and 67.9% among socially disadvantaged people in the United States (34). The prevalence of depressive disorders in diabetics is approximately 2-fold higher than the prevalence of depression in non-diabetics. Co-morbidity significantly worsens the prognosis of both illnesses and raises their mortality (6). Depression among individuals with DM is related with an increased risk of incident microvascular and macrovascular complications. A bidirectional relationship between depression and complications resulting from diabetes has been reported (35). Diabetic PHC patients with depression tended to have more severe physical symptoms, poorer self-care, and were demonstrated suboptimal adherence to prescribed care regiments (36).

Anxiety is an emotion with important implications in patients with T2DM. Elevated anxiety symptoms were found in 40% of diabetic patients (37). The prevalence rates of anxiety symptoms in PHC patients with T2DM were 30.5 to 40% in Malaysia (24, 25), and 38.3% in Saudi Arabia (29). The relationship between DM and anxiety has been reported to be bi-directional. A meta-analysis revealed that DM is associated with both elevated anxiety symptoms and anxiety disorders (38). Another meta-analysis found an association between baseline anxiety and incident DM (39). Lifetime anxiety symptoms have been shown to increase risk of hyperglycemia, contribute to more severe psychological symptoms, and sub-optimal self-management behavior among individuals with T2DM (40).

The American Diabetes Association notes that primary health care providers should consider evaluation for symptoms of DD, depression, and anxiety among PHC patients with T2DM using appropriate standardized and validated tools at their first visit, at periodic intervals, and when there is a change in illness, management, or life circumstance (41). Assessment of the complex psychological and emotional needs of people living with DM should be approached in a culture-sensitive method. Therefore, we used screening tools that were validated for use in Arabic-speaking countries and for PHC patients (42, 43).

The present study is motivated by the paucity of current research on the prevalence of DD, depression, and anxiety symptoms within the T2DM population of Egypt considering the COVID-19 context. As such, the primary goal of our study is to assess the prevalence and associated factors of DD, depression, and anxiety symptoms. We also investigate the relationship between symptoms of DD, depression, and anxiety among PHC patients with T2DM during the COVID-19 pandemic in Egypt. We hypothesized that T2DM patients experienced high levels of DD, depression, and anxiety symptoms during the COVID-19 pandemic; symptoms of DD have a statistically significant positive relationship with depressive and anxiety symptoms; depressive symptoms have a statistically significant positive relationship with anxiety symptoms; and a certain set of demographic and clinical characteristics of patients with T2DM are related to symptoms of DD, depression, and anxiety.

## Methods

### Design, Sampling, and Setting

Using a cross-sectional design, this study was carried out in eight rural primary care facilities at the Ismailia governorate, Egypt during the COVID-19 pandemic (between September 2020 and June 2021). A sample size of 369 was calculated using Epi InfoTM StatCalc version 7.2.4.0 (Centers for Disease Control and Prevention, Atlanta, GA, USA), given the data derived from a pilot study (*n* = 25). Calculation was based on the least prevalence obtained from our pilot study (4% for anxiety symptoms), 2% margin of error, and 95% level of confidence. The calculated sample size was further increased by 10% to compensate for the non-response. A convenience sampling of 406 patients with T2DM who met the eligibility criteria were interviewed during the study period. Patients were eligible if they were 18 years or older, had been diagnosed with T2DM for at least 1 year, and gave a written informed consent to participate. Three patients were excluded who had gestational diabetes or were not able to give their consent due to a serious mental illness or cognitive impairment. So, 403 participants were included in our study.

We obtained the ethical approval of this study from the Research Ethics Committee at the Faculty of Medicine, Suez Canal University, Ismailia, Egypt (Ref No. 4277/2020). All patients gave their written informed consent prior to their participation in this study.

### Tools and Measurements

Data collection was performed using face-to-face interviews with selected patients. Questionnaire included questions about sociodemographic, lifestyle and clinical characteristics: age, gender, marital status, occupation, family income, duration of diabetes, treatment for diabetes, diabetes-related long-term complications (e.g., cardiovascular, cerebrovascular, retinopathy, nephropathy, neuropathy, or peripheral vascular complications), smoking, alcohol drinking, and physical activity. Patients were also asked about history of COVID-19 (confirmed or suspected). Furthermore, the PAID was used to assess DD (9, 42, 44, 45), while the Patient Health Questionnaire-9 (PHQ-9) and the Generalized Anxiety Disorder Scale (GAD-7) were used to evaluate symptoms of depression and anxiety, respectively (46, 47).

The PAID scale consisted of 20 items, with a total score ranged from 0 to 100. Each item scored on a 5-point Likert scale ranging from 0 to 4, where 0 = not a problem, and 4 = serious problem. PAID total score was calculated by summing all items and multiplying it by 1.25. A higher score indicating greater DD, with a score of ≥40 indicating severe emotional distress (37–39). The Arabic version of the PAID has recently demonstrated to be a reliable and valid tool to screen DD in an Egyptian sample (42).

The PHQ-9 was used to evaluate the depressive symptoms where each item took a score from 0 to 3 (“not at all” to “nearly every day,” respectively). The total PHQ9 score was calculated as the sum of all items' scores, with a maximum score of 27. A total PHQ-9 score ≥10 showed a high sensitivity and specificity for major depression (46). The PHQ-9 was translated to Arabic with of a satisfactory validity and reliability (43).

The GAD-7 was used to evaluate the anxiety symptoms with each item taking a score from 0 to 3 (“not at all” to “nearly every day,” respectively). The sum of all items' score comprised a total GAD-7 score ranging from 0 to 21. A total GAD-7 score of 10 or higher was satisfactory sensitive and specific for GAD (47). An Arabic translation of GAD-7 is available with a satisfactory validity and reliability (43).

Body mass index (BMI) was calculated as the body weight (in kg) divided by the squared root of height (in meters), where patients were considered overweight if they had a BMI between 25 and 29.9, and obese if BMI ≥30. The world health organization has defined regular physical activity for adult people with chronic illness as engaging in at least 150 min or more of moderate-intensity aerobic activity per week; or at least 75 min or more of vigorous-intensity aerobic activity per week; or an equivalent combination of moderate- and vigorous-intensity activity weekly (48).

The most recent HbA1c values (<8 weeks prior to, or 12 weeks after interviewing the patient) and lipid profile [i.e., total cholesterol, high-density lipoprotein (HDL), low-density lipoprotein (LDL), and triglycerides] were obtained from patients' medical records. Good glycemic control was identified if HbA1c values were <7% in adult, or <7.5% in adults older than 65 years (41).

### Statistical Analysis

All procedures of data management and analyses were performed with the Statistical Package for the Social Sciences (SPSS) for Windows, version 25.0 (IBM Corporation, NY, USA). A significance level was set at 0.05 for all statistical analyses. Categorical variables were described as frequencies and percentages (%), while numeric variables were summarized as mean and standard deviation. Associations between categorical variables were investigated for statistical significance with Chi-square test or Fischer's exact test as indicated. Graphs were created with GraphPad Prism (version 8.0.0 for Windows, GraphPad Software, San Diego, California USA, www.graphpad.com). Multiple hierarchical logistic regression models were used to identify the predictors of DD, depression, and anxiety symptoms among the studied patients. Independent variables were entered in the model as blocks. Three blocks were identified: the first block involved sociodemographic variables, second block included lifestyle and general health variables, while the third block included diabetes-related variables. Improvement in the predictive power of the consecutive models was identified by calculating the change in −2 log likelihood (−2LL) and was tested for statistical significance using the chi-square distribution (where the degree of freedom was the difference in the number of parameters in each model). Also, the change in the R-square and the predictive accuracy were reported for each model. Odds ratio (OR) and 95% confidence interval (CI) was reported for each independent variable in the models.

## Results

**Figure 1:**
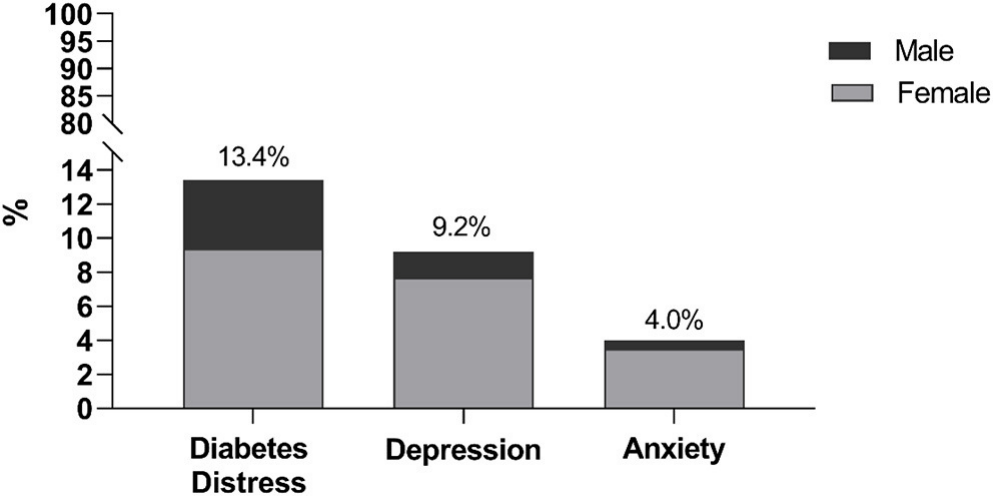
Diabetic patients with scores suggestive of severe diabetes distress, major depression, and generalized anxiety disorder.

This study involved 403 patients with T2DM with a mean age of 46 years (±11.5; range: 19–80 years), and 59.1% were female. Demographic, lifestyle, and health-related characteristics are presented in Tables 1, 2. Figure 1 shows that 13.4% (95% CI: 10.1–16.7) of diabetic patients had a PAID score indicating severe DD, while 9.2% (95% CI: 6.4–12.0%) had a PHQ-9 score suggestive of major depression. Only 4.0% (95% CI: 2.1–5.9) of diabetic patients had a GAD-7 score suggestive for generalized anxiety. Furthermore, female patients comprised the majority of patients who had symptoms of DD, depression, and anxiety (70.4, 83.8, and 87.5%, respectively).

**Table 1:** Distribution of diabetic patients according to their sociodemographic characteristics (*N* = 403).

		**No. (Row %)**					
**Characteristics**	**All Participants** **No. (Column %)** ***N* = 403**	**Diabetes** **Distress *n* = 54**	***p*-value**	**Depression** ***n* = 37**	***p*-value**	**Anxiety *n* = 16**	***p*-value**
**Age** (years)							
Less than 40 years	103 (25.6%)	6 (5.8%)	<0.001^*^	2 (1.9%)	<0.001^*^	2 (1.9%)	<0.001^*^
40–59	222 (55.1%)	16 (7.2%)		13 (5.9%)		3 (1.4%)	
60+	78 (19.4%)	32 (41.0%)		22 (28.2%)		11 (14.1%)	
**Gender**							
Male	165 (40.9%)	16 (9.7%)	0.069	6 (3.6%)	0.002^*^	2 (1.2%)	0.018^*^
Female	238 (59.1%)	38 (16.0%)		31 (13.0%)		14 (5.9%)	
**Marital status**							
Single	19 (4.7%)	0	<0.001^*^	0	<0.001^*^	0	**0.002** ^*^ ^F^
Married	307 (76.2%)	31 (10.1%)		18 (5.9%)		7 (2.3%)	
Divorced/widow	77 (19.1%)	23 (29.9%)		19 (24.7%)		9 (11.7%)	
**Education level**							
Illiterate	87 (21.6%)	35 (40.2%)	<0.001^*^	28 (32.2%)	<0.001^*^	13 (14.9%)	**<0.001** ^*^ ^F^
Less than secondary	15 (3.7%)	1 (6.7%)		1 (6.7%)		2 (13.3%)	
Secondary	239 (59.3%)	17 (7.1%)		7 (2.9%)		1 (0.4%)	
University and above	62 (15.4%)	1 (1.6%)		1 (1.6%)		0	
**Occupation**							
None	232 (57.6%)	50 (21.6%)	**0.000** ^*^ ^F^	36 (15.5%)	<0.001^*^	16 (6.9%)	**0.020** ^*^ ^F^
Manual work and sales	72 (17.9%)	4 (5.6%)		0		0	
Clerical or administrative work	29 (7.2%)	0		1 (3.4%)		0	
Professionals and their associates	58 (14.4%)	0		0		0	
Business owners and freelancers	12 (3.0%)	0		0		0	
**Family income**							
Insufficient	94 (23.3%)	33 (35.1%)	<0.001^*^	25 (26.6%)	<0.001^*^	10 (10.6%)	**<0.001** ^*^ ^F^
Sufficient	309 (76.7%)	21 (6.8%)		12 (3.9%)		6 (1.9%)	

* *Statistically significant p-value at p < 0.05*.

F *Fisher's exact test*.

*Bold value indicates the significant findings*.

*The table shows only the groups positive for diabetes distress, depression, and anxiety symptoms. All statistical significances were tested by comparing groups positive for the study outcomes (i.e., diabetes distress, depression and anxiety symptoms) to those who were negative for these outcomes*.

**Table 2:** Patients' lifestyle and health-related characteristics (*N* = 403).

		**No. (Row %)**					
**Characteristics**	**All Participants** **No. (Column %)** ***N* = 403**	**Diabetes** **Distress *n* = 54**	***p*-value**	**Depression** ***n* = 37**	***p*-value**	**Anxiety *n* = 16**	***p*-value**
**Body Mass Index** (kg/m**^2^**), Mean (SD)	403 (100.0%)	29.6 (8.0)	0.613^M^	30.6 (9.1)	0.326^M^	34.0 (11.5)	0.113^M^
Normal	99 (24.6%)	17 (17.2%)	0.023^*^	12 (12.1%)	0.036^*^	5 (5.1%)	0.041^*^ **^F^**
Overweight	166 (41.2%)	13 (7.8%)		8 (4.8%)		2 (1.2%)	
Obese	138 (34.2%)	24 (17.4%)		17 (12.3%)		9 (6.5%)	
**Sex-specific waist circumference** (cm)							
Men, mean (SD)	91.6 (13.1)	89.9 (19.4)	0.219^M^	75.2 (8.7)	0.001^*^ **^M^**	70.0 (7.1)	0.019^*^ **^M^**
Women, mean (SD)	92.2 (17.1)	94.3 (18.1)	0.373^M^	100.8 (18.9)	0.002^*^ **^M^**	107.6 (21.1)	0.004^*^ **^M^**
Overall, mean (SD)	91.9 (15.5)	93.0 (18.4)	0.282^M^	96.7 (20.0)	0.089^M^	102.9 (23.6)	**0.041** ^*^ ^M^
**Life-style characteristics**							
Ever cigarette smoking	130 (32.3%)	14 (10.8%)	0.285	6 (4.6%)	0.028^*^	2 (1.5%)	0.084
Alcohol drinking	2 (0.5%)	0	1.000^F^	0	1.000^F^	0	1.000^F^
Physical inactivity	103 (25.6%)	34 (33.3%)	<0.001^*^	27 (26.2%)	<0.001^*^	13 (12.6%)	**<0.001** ^*^ ^F^
**Duration of diabetes**							
Less than 5 years	143 (35.5%)	6 (4.2%)	<0.001^*^	3 (2.1%)	<0.001^*^	0	<0.001^*^
5–10 years	161 (40.0%)	17 (10.6%)		10 (6.2%)		5 (3.1%)	
More than 10 years	99 (24.6%)	31 (31.3%)		24 (24.2%)		11 (11.1%)	
**Type of antidiabetic medications**							
Oral hypoglycemics	272 (67.5%)	20 (7.4%)	<0.001^*^	13 (4.8%)	<0.001^*^	4 (1.5%)	<0.001^*^
Insulin-containing regimens	131 (32.5%)	34 (26.0%)		24 (18.3%)		12 (9.2%)	
**Number of diabetic complications**							
None	139 (34.5%)	6 (4.3%)	<0.001^*^	1 (0.7%)	<0.001^*^	0	0.002^*^
Single	101 (25.1%)	7 (6.9%)		6 (5.9%)		3 (3.0%)	
Two or more	163 (40.4%)	41 (25.2%)		30 (18.4%)		13 (8.0%)	
**Type of complications**							
Retinopathy	155 (38.5%)	40 (25.8%)	<0.001^*^	29 (18.7%)	<0.001^*^	13 (8.4%)	<0.001^*^
Nephropathy	95 (23.6%)	38 (40.0%)	<0.001^*^	29 (30.5%)	<0.001^*^	13 (13.7%)	**<0.001** ^*^ ^F^
Peripheral neuropathy	208 (51.6%)	46 (22.1%)	<0.001^*^	32 (15.4%)	<0.001^*^	15 (7.2%)	0.001^*^
Autonomic neuropathy	4 (1.0%)	0	1.000^F^	0	1.000^*^^F^	0	1.000^F^
Coronary or cerebrovascular	2 (0.5%)	1 (50.0%)	0.250^F^	1 (50.0%)	0.175^F^	1 (50.0%)	0.078**^F^**
Peripheral vascular	126 (31.3%)	37 (29.4%)	**<0.001** ^*^ ^F^	29 (23.0%)	<0.001^*^	13 (10.3%)	<0.001^*^
**Number of chronic comorbidities**							
None	300 (74.5%)	17 (5.7%)	<0.001^*^	11 (3.7%)	<0.001^*^	2 (0.7%)	**<0.001** ^*^ ^F^
Single	65 (16.1%)	17 (26.2%)		9 (13.8%)		2 (3.1%)	
Two or more	38 (9.4%)	20 (52.6%)		17 (44.7%)		12 (31.6%)	
**Type of chronic comorbidities**							
Hypertension	89 (22.1%)	34 (38.2%)	<0.001^*^	24 (27.0%)	<0.001^*^	13 (14.6%)	**<0.001** ^*^ ^F^
Dyslipidemia	36 (8.9%)	19 (52.8%)	**<0.001** ^*^ ^F^	14 (38.9%)	**<0.001** ^*^ ^F^	10 (27.8%)	**<0.001** ^*^ ^F^
Others **^a^**	23 (5.5%)	7 (31.8%)	**0.018** ^*^ ^F^	9 (40.9%)	**<0.001** ^*^^F^	6 (27.3%)	**<0.001** ^*^ ^F^
**HbA1C%**, mean ± SD	7.8 ± 0.7	8.7 ± 1.2	<0.001^*^	8.9 ± 0.8	<0.001^*^	8.2 ± 0.7	<0.001^*^
**Glycemic control**							
Controlled	30 (7.4%)	2 (6.7%)	0.204^F^	1 (3.3%)	0.341^F^	0	0.621^F^
Uncontrolled	373 (92.6%)	52 (13.9%)		36 (9.7%)		16 (4.3%)	
**Lipid Profile, mean** **±SD**							
Total cholesterol	196.5 ± 16.7	204.8 ± 19.8	0.001^*^	209.7 ± 27.2	0.004^*^	208.4 ± 33.5	0.005^*^
HDL	63.6 ± 9.2	62.0 ± 8.7	0.189		0.091	61.6 ± 11.5	0.399
LDL	67.1 ± 18.7	79.8 ± 23.8	<0.001^*^	61.1 ± 9.2	<0.001^*^	92.6 ± 20.6	<0.001^*^
Triglycerides	93.4 ± 37.9	119.0 ± 39.5	<0.001^*^	87.6 ± 27.3 135.9 ± 69.2	<0.001^*^	124.2 ± 41.3	0.001^*^
**Family history of diabetes**							
No	92 (22.8%)	6 (6.5%)	0.027^*^	6 (6.5%)	0.315^F^	2 (2.2%)	0.542^F^
Yes	311 (77.2%)	48 (15.4%)		31 (10.0%)		14 (4.5%)	
**History of COVID-19**							
PCR-confirmed diagnosis	14 (3.5%)	2 (14.3%)	1.000^F^	0	0.628^F^	0	1.000^F^
Clinically-suggestive diagnosis	15 (3.7%)	2 (13.3%)	1.000^F^	2 (13.3%)	0.638^F^	2 (13.3%)	0.115^F^

a *Other chronic diseases included 11 patients with gastrointestinal & Liver, six patients with peripheral venous, one patient with end-stage-renal disease (ESRD), one patient with a neurological disease, and three patients with musculoskeletal diseases*.

F *Fisher's exact test*;

M *. Mann-Whitney test*.

* *Statistically significant p-value at p < 0.05*.

*SD, standard deviation; HDL, High-density lipoprotein; LDL, Low-density lipoprotein*.

*Bold value indicates the significant findings*.

*The table shows only the groups positive for diabetes distress, depression, and anxiety symptoms. All statistical significances were tested by comparing groups positive for the study outcomes (i.e., diabetes distress, depression and anxiety symptoms) to those who were negative for these outcomes*.

Table 1 shows that most of the diabetic patients were married, had completed their secondary or higher education, not working (including housewives and retired), and had sufficient family income (76.2, 74.7, 57.6, and 76.7%, respectively). Symptoms of depression and anxiety were significantly associated with all demographic variables while DD was significantly associated with all demographic characteristics except for gender. DD, depression, and anxiety symptoms were frequent among older patients (≥60 years), female, unmarried (single, divorced or widowed), illiterate, not working (including housewives and retired) and patients with insufficient family income.

Table 2 shows that DD associated significantly with overweight and obesity, physical inactivity, longer duration of diabetes (>10 years), insulin-containing medications, multiple diabetic complications (particularly retinopathy, nephropathy, peripheral neuropathy, and peripheral vascular diseases), multiple chronic comorbidities, the higher levels of HbA1c, total cholesterol, LDL and triglycerides, and the existence of family history of DM. Likewise, depressive and anxiety symptoms concurred in having significant associations with overweight and obesity, sex-specific waist circumference, physical inactivity, longer duration of diabetes, insulin-containing medications, multiple diabetic complications, multiple chronic comorbidities, and the higher levels of HbA1c, total cholesterol, LDL, and triglycerides. However, the history of COVID-19 and the glycemic control did not show significant association with any of the study outcomes (i.e., symptoms of DD, depression, or anxiety).

**Figure 2:**
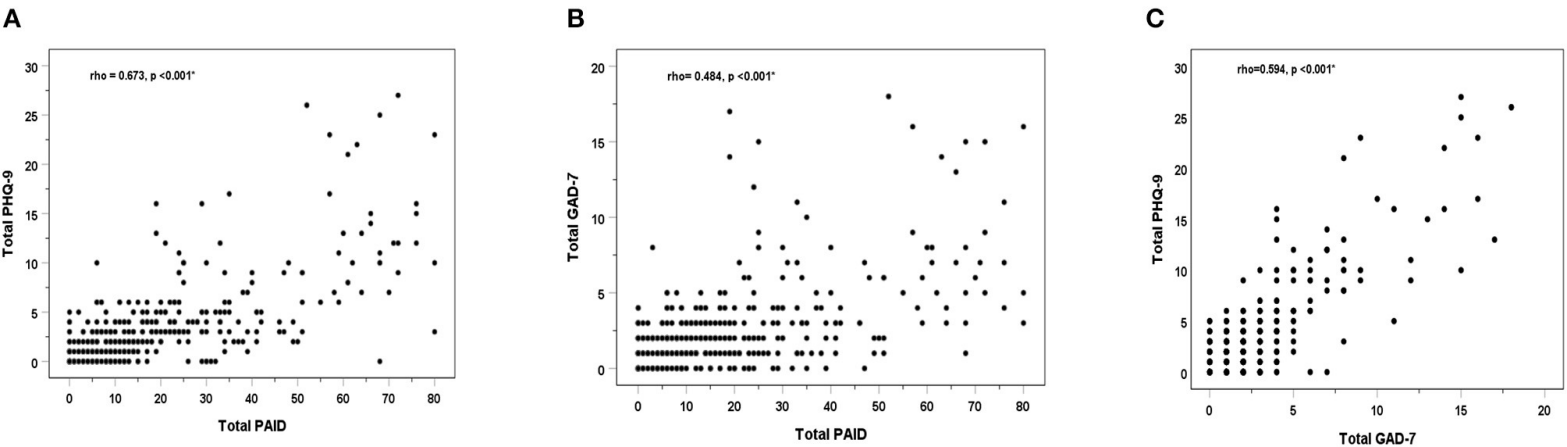
Spearman's Correlations (rho) between total scores of the study outcome variables: the PAID for diabetes distress, the PHQ-9 for depression, and the GAD-7 for anxiety: PAID vs. PHQ-9 **(A)**, PAID vs. PHQ-9 **(B)**, and GAD-7 vs. PHQ-9 **(C)**. * Statistically significant correlation coefficient at *p* < 0.05.

Figure 2 shows that the total PAID score for symptoms of DD were positively correlated with both the total PHQ-9 score for depressive symptoms and the total GAD-7 score for anxiety symptoms (rho: 0.673 and 0.484, respectively, *p* < 0.001). Likewise, the PHQ-9 and the GAD-7 showed a significant, moderate, and positive correlation (rho: 0.594, *p* < 0.001).

Table 3 displays results of the hierarchical logistic regression analyses for symptoms of DD, depression, and anxiety. Model 1 included only the potential sociodemographic predictors (i.e., block 1) and showed a statistically significant change in the −2LL from the baseline model (containing only the constant). Model 1 accounted for 37.5, 36.5, and 27% of the variation in symptoms of diabetic distress, depression, and anxiety, respectively. By adding the block 2 (i.e., the life-style and general health variables), the predictive power of the DD, depression and anxiety models was improved as indicated by the significant change in the model −2LL and the increasing R-square. Likewise, adding the block 3 (i.e., the diabetes-related variables) significant improved the predictive power of each model and increased the model R-square. In the last model for DD (model 3), the only significant predictors were being married, illiterate, not working, living with insufficient income, and having multi-comorbidities. However, the last model for depressive symptoms showed that the higher HbA1c levels and the PAID score were the only significant predictors, and the last model for anxiety revealed that multi-comorbidities, the elevated HbA1c levels and the PAID score were the only significant predictors. The odds ratios of significant predictors and its 95% confidence intervals are presented in Table 3.

**Table 3:** Hierarchical logistic regression models for prediction of diabetes distress, depression, and anxiety symptoms among diabetic patients (*N* = 403).

**Predictors**	**Diabetes Distress**, ***n*****=** **54**			**Depression** ***n*** **=** **37**			**Anxiety**, ***n*** **=** **16**		
	**Model 1**	**Model 2**	**Model 3**	**Model 1**	**Model 2**	**Model 3**	**Model 1**	**Model 2**	**Model 3**
***Block 1: Sociodemographic variables***									
Age ≥60 years	2.79 (0.98–7.92)	2.16 (0.65–7.17)	2.09 (0.60–7.23)	1.48 (0.46–4.73)	0.98 (0.26–3.68)	0.28 (0.05–1.62)	2.98 (0.60–14.7)	1.10 (0.15–7.93)	0.39 (0.03–5.20)
Female	0.82 (0.30–2.27)	1.41 (0.31–6.49)	1.37 (0.29–6.51)	1.68 (0.51–5.59)	2.45 (0.41–14.6)	6.76 (0.36–127.0)	3.77 (0.74–19.1)	3.48 (0.28–43.7)	10.1 (0.32–325.5)
Married	2.14 (0.79–5.79)	**3.19 (1.03–9.90)** ^*^	**3.63 (1.12–11.7)** ^*^	1.06 (0.36–3.08)	1.28 (0.38–4.29)	0.46 (0.09–2.24)	1.29 (0.30–5.64)	1.17 (0.21–6.65)	0.56 (0.06–5.37)
Illiterate	**3.90 (1.66–9.19)** ^*^	**3.60 (1.44–9.01)** ^*^	**3.63 (1.37–9.62)** ^*^	**4.85 (1.70–13.9)** ^*^	**4.04 (1.33–12.2)** ^*^	1.70 (0.33–8.75)	7.85 (1.54–40.1)^*^	5.50 (1.00–30.3)^*^	7.31 (0.55–97.1)
Not working	**5.74 (1.45–22.7)** ^*^	**6.09 (1.44–25.7)** ^*^	**5.91 (1.34–26.1)** ^*^	8.36 (0.89–78.3)	7.86 (0.82–75.1)	2.03 (0.13–30.9)	– ^a^	– ^a^	– ^a^
Insufficient income	**2.86 (1.34–8.07)** ^*^	**2.76 (1.26–6.05)** ^*^	**2.47 (1.09–5.64)** ^*^	**2.90 (1.17–7.20)** ^*^	**2.72 (1.05–7.04)** ^*^	1.29 (0.37–4.49)	1.32 (0.37–4.69)	1.04 (0.25–4.26)	0.42 (0.07–2.52)
***Block 2: Life-style and general health***									
Overweight/ obese		0.51 (0.22–1.20)	0.51 (0.21–1.21)		0.67 (0.25–1.83)	0.86 (0.21–3.49)		1.24 (0.28–5.43)	2.52 (0.27–23.7)
Cigarette smoker		2.09 (0.51–8.62)	2.11 (0.49–9.20)		1.61 (0.28–9.12)	1.52 (0.08–29.8)		1.18 (0.09–15.07)	2.61 (0.07–97.3)
Physically inactive		1.41 (0.56–3.59)	1.42 (0.52–3.86)		1.42 (0.45–4.43)	1.16 (0.24–5.65)		1.23 (0.17–8.91)	1.79 (0.14–23.3)
Multiple comorbidities (≥2)		**3.03 (1.16–7.87)** ^*^	**2.90 (1.04–8.08)** ^*^		**3.72 (1.36–10.1)** ^*^	1.08 (0.26–4.47)		15.5 (3.42–69.9)^*^	**8.22 (1.16–58.3)** ^*^
***Block 3: Diabetes-related variables***									
Duration of diabetes ≥10 years			0.95 (0.33–2.65)			1.67 (0.37–7.54)			0.48 (0.06–4.01)
Insulin-containing medications			1.58 (0.64–3.95)			1.47 (0.37–5.87)			3.00 (0.43–20.7)
Multiple diabetic complications (≥2)			1.04 (0.41–2.62)			2.33 (0.42–13.0)			1.42 (0.10–20.2)
HA1C %			1.21 (0.71–2.05)			4.85 (1.95–12.1)^*^			7.34 (2.22–24.2)^*^
Total cholesterol (mg/dl)			0.99 (0.97–1.02)			0.99 (0.95–1.02)			0.97 (0.93–1.01)
Triglycerides (mg/dl)			1.00 (0.99–1.01)			0.99 (0.98–1.02)			0.98 (0.96–1.00)
PAID-20			-			1.09 (1.06–1.13)^*^			1.07 (1.02–1.11)^*^
Δ−2LL (*df, p*-value)	91.75 (6, <0.001^*^)	11.90 (4, 0.018^*^)	1.63 (6, 0.951)	71.33 (6, 0.000^*^)	10.43 (4, 0.034^*^)	58.99 (7, 0.000^*^)	30.71 (5, 0.000^*^)	17.50 (4, 0.002^*^)	22.98 (7, 0.002^*^)
Nagelkerke R-Square	0.375	0.417	0.423	0.365	0.413	0.663	0.27	0.415	0.596
Δ R^−^Square	0.375	0.042	0.006	0.365	0.048	0.25	0.27	0.145	0.181
Predictive accuracy %	87.8%	89.8%	89.5%	96.3%	91.5%	94.8%	96.3%	96.3%	97.3%

*−2LL,−2 Log Likelihood; df, degree of freedom*.

* *Statistically significant p-value at p < 0.05*.

***Reference categories for categorical variables, respectively as appeared in the table**: age <60 years, male, not-married (including single, widow, or divorced), working, normal BMI, non-smoker, physically active, less than two comorbidities, duration of diabetes <10 years, oral hypoglycemics, and less than two diabetic complications*.

a *Variable excluded due to insufficient responses in patients with anxiety*.

*Bold value indicates the significant findings*.

## Discussion

According to the study findings, approximately one in seven, one in 10 and one in 25 primary care patients with T2DM in the rural area of Ismailia governorate in Egypt were suffering from symptoms of severe DD, major depression and anxiety, respectively. Several sociodemographic and clinical characteristics were identified to be associated with these findings at a different degree.

The prevalence of DD in the current study was higher than what was reported in a meta-analysis by Perrin et al. (11) and in primary care patients in Netherland, USA, Germany, and Thailand (12, 13, 15, 16), but less than the prevalence in Saudi Arabia and Greece (17, 18). These marked discrepancies between different study findings could be attributed to many reasons including cultural, social, demographic, and health-related characteristics of the study populations as well as tools of DD assessment. Moreover, Perrin et al. meta-analysis had an extremely high level of heterogenicity and asymmetrical funnel plot suggesting larger representation of studies with more prevalent DD. It is also important to note that the American, Dutch, Germany and Thai studies were carried out before COVID-19 era (12, 13, 15, 16).

Just <10% of our study sample had symptoms of major depression (PHQ-9 ≥10). Prevalence rates of comorbid depression in diabetes was variably reported with a range of 2–88% with a world-pooled prevalence of 28% (49). According to Lloyd et al., social and cultural factors influence depression occurrence leading to different prevalence rates of depression-related conditions among individual countries and within different communities and ethnicities in the same country (50). Furthermore, cultural meaning of depression could be expressed differently between different populations. For example, a focus group interview of patients with T2DM living in the United Kingdom found that patients with T2DM who were from Bangladeshi and Pakistani background often expressed symptoms of depression in a somatic way (51). In fact, it is increasingly recognized that primary care patients throughout the world express depression with somatic manifestations irrespective of their cultural background (52). Therefore, a qualitative assessment of depression burden in our population is needed to complement the study findings.

The least reported comorbid psychiatric problem in patients with T2DM in this study was anxiety symptoms with only 4% of the study sample having a minimum score of 10 on the GAD questionnaire. Previous literature also showed higher rates of anxiety disorders among patients with T2DM ranging between 30 and 40%. However, these studies used different tools for anxiety assessment which makes direct comparison to our study findings difficult (24, 25, 32, 37). Nevertheless, Smith et al. concluded that DM is weakly and positively associated with anxiety symptoms and anxiety disorders with a pooled OR of 1.25 (CI: 1.10–1.39) with low levels of statistical heterogeneity (38).

Complex interactions between DM and social as well as cultural dynamics are likely to affect the way patients experience illness and health (50). Our study did not find an association between gender and higher risk for developing DD which is consistent with Kamrul-Hasan et al. findings (53). However, bivariant analysis showed that females had higher risk for developing depression and anxiety although that was not confirmed with subsequent multivariant analysis. Previous studies demonstrated an association between female gender and DD (11, 13, 17, 19, 54, 55), and female gender and comorbid depression and anxiety with T2DM (19, 54). It is postulated that socio-cultural and biological factors may be implicated for this gender difference increasing female patients' vulnerability to life events and affecting their coping skills (56). On the other hand, males appear less likely to seek medical advice or express distress leading to spuriously lower prevalence rates of emotional difficulties (57).

Regarding age, bivariant analysis of this study suggested that symptoms of DD, depression, and anxiety occurred more frequently with increasing age. However, multivariant analysis did not find an association between age with either DD, depression or anxiety symptoms. Similarly, a Malaysian cross-sectional study on PHC patients with T2DM did not find a statistical association between age and prevalence of depressive and anxiety symptoms (24). on the contrary, another two studies in Saudi Arabia and Australia reported slightly lower rates of psychological disorders in older patients with DM compared to patients with younger age (29, 55). These differences could reflect unmet needs of older patients with T2DM in our population and should be followed by further research to identify the underlying causes of this outcome.

The current study's bivariant analysis showed that illiteracy, insufficient family income, unemployment, and being divorced or widowed were associated with higher prevalence of DD, depression, and anxiety symptoms. Study findings from other countries also showed similar association although not all associations were statistically significant (53, 58). Consequently, optimizing management of patients with DM does not only require a multidisciplinary team of healthcare workers, but it also involves synergistic multi-dimensional care plan of the surrounding environment.

Lifestyle factors like physical inactivity and obesity were associated with higher rates of diabetes associated mental health disorders in our study. This association was also confirmed in a longitudinal study that found that persistent depressive symptoms at 5 years were significantly associated with worse compliance with dietary and physical activity recommendations compared to patients with diabetes without depressive symptoms (59). Moreover, increased depression scores overtime predicted lesser adherence rates to dietary and exercise recommendations (60). Therefore, addressing psychological needs of patients with DM could help to improve patients' self-care and quality of life.

COVID-19 pandemic exerted a tremendous pressure on both patients and healthcare providers with unknown long-term consequences. Although we did not find a significant association between history of COVID-19 infection and the prevalence of mental health disorders among diabetic patients in our study, an earlier longitudinal study in Australia found that COVID-19 lockdown restrictions had negative impact on patients with T2DM quality of life and physical activity levels without affecting generalized anxiety and depressive symptoms prevalence (61). Therefore, ensuring access to mental health services for vulnerable patients during this unprecedent time cannot be overrated.

In the current study, diabetes duration, complications and treatment regimen were all associated with increased risks for comorbid DD, depression, and anxiety symptoms, which is consistent with previous studies (53, 55, 58). However, it is difficult to interpret potential risk factors for mental health disorders in T2DM as these factors often coexist and interact with each other. Although using insulin was associated with increased rate of diabetes-related psychological disorders (in bivariant analysis), this might be confounded by the fact patients with T2DM receiving insulin-based regimens might have had diabetes for a longer period and/or have higher rate of comorbid diseases or diabetes-related complications.

By using multiple logistic regression, we found that the only predictors for DD were social factors (namely being married, illiterate, having insufficient income) and having multiple co-morbidities. Nevertheless, DD itself as well as HbA1c level were predictors for depressive and anxiety symptoms in patients with T2DM. The intercorrelation between social factors, health-related variables, DD, depression, and anxiety seem to go into a continuous cycle with complex interactions that necessitate a holistic patient-centered approach in order to break this cycle.

Our study provided some of the early evidence on the burden of three important psychological disorders in people with T2DM who are managed in the PHC centers in the rural area of Ismailia in Egypt. However, this observational study is subjected to limitations. First, due to the cross-sectional nature of the study, it is not possible to identify causality between variables. Therefore, a further longitudinal study is needed to reveal the strength and direction of any potential association. Second, as we did not have baseline data on the burden on mental health disorders among patients with T2DM in our population, we could not assess the impact of COVID-19 pandemic on our study participants. Third, lack of randomization limited the ability to generalize the results. Fourth, the use of western methods to identify psychological disorders in non-western countries could be questionable. However, all the scales that we used (the PAID, the PHQ-9, and the GAD-7) were validated for use in Arabic language. Nevertheless, developing culturally sensitive screening tools could help in better assessment of psychological disorders in non-English speaking patients with DM.

## Conclusions

Our study found DD is more prevalent than depressive and anxiety symptoms in adults with T2DM managed in the PHC facilities in the rural area of Ismailia in Egypt during the COVID-19 pandemic. DD and HbA1c level were associated with depressive and anxiety symptoms in this population of patients. Although psychosocial assessment is important for all patients with T2DM, our study findings suggest that PHC providers should pay closer attention to females, elderly, patients suffering from DM for a longer time, those with multiple comorbidities and/or chronic diabetes complications. As multiple sociodemographic and clinical factors were identified to be linked with psychological conditions in patients with T2DM, it is important to utilize multidisciplinary teams to achieve holistic patient-centered care. Further studies are necessary to better understand the long-term psychological consequences of the COVID-19 pandemic on patients with T2DM.

## Data Availability Statement

The original contributions presented in the study are included in the article/supplementary material, further inquiries can be directed to the corresponding author/s on reasonable request.

## Ethics Statement

The studies involving human participants were reviewed and approved by Research Ethics Committee at the Faculty of Medicine, Suez Canal University, Ismailia, Egypt (Ref No. 4277/2020). The patients/participants provided their written informed consent to participate in this study.

## Author Contributions

HS commenced the idea of this research. HS, AF, AJ, JS, and AS drafted this manuscript. SM collected the data of this research. AF and SE analyzed these data. SE, MM, JS, and SM critically reviewed this manuscript. SM and HS supervised this research. All authors designed this research and approved the final manuscript.

## Conflict of Interest

AJ was employed by Hamad medical Corporation. The remaining authors declare that the research was conducted in the absence of any commercial or financial relationships that could be construed as a potential conflict of interest.

## Publisher's Note

All claims expressed in this article are solely those of the authors and do not necessarily represent those of their affiliated organizations, or those of the publisher, the editors and the reviewers. Any product that may be evaluated in this article, or claim that may be made by its manufacturer, is not guaranteed or endorsed by the publisher.

## Abbreviations

−2LL: −2 Log Likelihood
BMI: Body Mass Index
df: degree of freedom
CI: Confidence interval
DD: Diabetes distress
DM: Diabetes mellitus
GAD-7: Generalized Anxiety Disorder Scale 7
HbA1c: Glycated hemoglobin
HDL: High-density lipoprotein
IQR: Interquartile range
LDL: Low-density lipoprotein
OR: Odds ratio
PAID: Problem Areas in Diabetes
PHC: Primary healthcare
PHQ-9: Patient Health Questionnaire 9
Rho: Spearman's Rank-Order Correlation
SD: Standard deviation
SPSS: Statistical Package for the Social Sciences
T2DM: Type 2 diabetes mellitus
WHO: World Health Organization.
